# Genetic ablation of the adult Leydig cell lineage reveals stem Leydig cells and a peritubular regenerative niche in mice

**DOI:** 10.1210/endocr/bqag113

**Published:** 2026-10-09

**Authors:** Laurie Boudreau, Nicholas M Robert, Jacques J Tremblay

**Affiliations:** Reproduction, Mother and Child Health, Centre de recherche du centre hospitalier universitaire de Québec—Université Laval, Québec City, QC G1V 4G2, Canada; Reproduction, Mother and Child Health, Centre de recherche du centre hospitalier universitaire de Québec—Université Laval, Québec City, QC G1V 4G2, Canada; Reproduction, Mother and Child Health, Centre de recherche du centre hospitalier universitaire de Québec—Université Laval, Québec City, QC G1V 4G2, Canada; Department of Obstetrics, Gynecology, and Reproduction, Faculty of Medicine, Centre de recherche en Reproduction, Développement et Santé Intergénérationnelle, Université Laval, Québec City, QC G1V 0A6, Canada

**Keywords:** Leydig cells, Leydig cell regeneration, *Insl3* ^iCre^ mice, peritubular myoid cells, stem Leydig cells, diphtheria toxin

## Abstract

Differentiated Leydig cells (LCs) produce testosterone and INSL3, 2 hormones essential for male sex differentiation and fertility. In mice, the developmental origin of adult Leydig cells (ALCs) and the presence of stem Leydig cells (SLCs) in adulthood remain unresolved. To investigate this, we used the Leydig cell-specific *Insl3*^iCre^ line combined with *Rosa26*^lox-STOP-lox-iDTR/lox-STOP-lox-TdTomato^ to genetically ablate the committed LC lineage in adult mice. These mice displayed a normal phenotype without diphtheria toxin (DT). Following DT administration, TdTomato-positive and CYP17A1-positive LCs were eliminated within 4 to 7 days, leading to marked reductions in testosterone levels and androgen-dependent organ weights. Activated Caspase 3 confirmed apoptosis of targeted Leydig cells during the acute ablation phase. This was accompanied by transient macrophage recruitment as shown by F4-80 immunofluorescence, consistent with their involvement in clearing apoptotic Leydig cells. Importantly, COUP-TFII-positive/αSMA-negative/CYP17A1-negative/TdTomato-negative cells persisted adjacent to peritubular and perivascular myoid cells, identifying a candidate *Insl3*-negative precursor population. Newly emerging TdTomato-positive cells subsequently appeared predominantly around seminiferous tubules and, to a lesser extent, blood vessels, marking the onset of Leydig cell regeneration. Testosterone levels progressively recovered as these cells acquired CYP17A1 expression and populated the interstitial compartment. By 65 days post-DT, the ALC population and endocrine function were fully restored. These findings provide strong functional evidence for adult SLCs capable of repopulating the LC lineage after complete ablation and identify the peritubular compartment as a major regenerative niche. This inducible LC-depletion mouse model provides a powerful tool to investigate SLC biology and the mechanisms governing LC differentiation and testicular endocrine homeostasis.

Located in the interstitial space between the seminiferous tubules of the mammalian testis, Leydig cells are specialized endocrine cells that produce 2 major hormones, androgens (mainly testosterone) and insulin-like 3 (INSL3). INSL3 and androgens are indispensable for the development and functional maintenance of the male reproductive system during both fetal and postnatal life (reviewed in Refs. (1-3)). However, their production is mediated by distinct populations of Leydig cells at different developmental stages: fetal Leydig cells (FLCs) and adult Leydig cells (ALCs).

FLCs are responsible for the synthesis of androgens and INSL3 required for masculinization of the male urogenital tract during fetal development (reviewed in Refs. (4, 5)). Despite their essential role, the majority of FLCs undergo regression soon after birth (reviewed in Refs. (2, 4, 5)), with only ∼1% persisting in the postnatal mouse testis (6). FLCs are therefore not considered to contribute substantially to the ALC population. Although the precise origin of ALCs remains incompletely understood, several studies suggest that FLCs and ALCs may arise from a common pool of progenitor cells present in the fetal testis (reviewed in Refs. (2, 7)). These precursor cells, or stem Leydig cells (SLCs), are thought to appear around embryonic day 16.5 (E16.5) in mice (8) and remain quiescent until approximately postnatal day 5-7 (P5-P7) (9). Postnatal ALC development is generally described as a sequential differentiation process in which SLCs give rise to committed progenitor Leydig cells, followed by immature and ultimately mature ALCs. This sequence has been characterized most extensively in rats, but an analogous progression occurs in mice, with species-specific differences in timing and steroidogenic properties of the individual stages ((10) and reviewed in Refs. (11-13)). In mice, commitment to the adult Leydig lineage begins during the first postnatal week, consistent with the onset of *Insl3*^iCre^ activity around P5 in our previously characterized lineage-tracing model (9). In adulthood, ALCs exhibit minimal proliferation and low turnover, allowing them to remain functional throughout life (6, 14). However, their capacity for steroid production gradually declines with age, particularly in humans, resulting in reduced testosterone levels and potentially contributing to age-related hypogonadism (reviewed in Refs. (15, 16)).

In most mammalian species, including the mouse (17), a subset of SLCs persists in the adult testis as a largely quiescent population that serves as a reservoir, capable of differentiation into ALCs in response to physiological or experimental stimuli. Their ability to regenerate a functional ALC population has been primarily demonstrated in experimental models, especially in rats treated with the cytotoxic agent ethane dimethanesulfonate (EDS). Although associated with some systemic toxicity (18-25), EDS efficiently and selectively ablates mature, but not immature, ALCs in rats and this is followed by the appearance of a new generation of functional ALCs within a few weeks (26-32). However, EDS is inefficient in mice (33, 34) requiring high doses to achieve partial depletion, which in turn causes substantial systemic effects (35, 36).

To overcome the limitations of EDS-based Leydig cell ablation in murine models, the improved diphtheria toxin receptor (iDTR) system offers a powerful alternative. When paired with a Cre-Lox conditional strategy that restricts iDTR expression specifically to Leydig cells, this approach enables precise temporal control of cell ablation through diphtheria toxin (DT) administration. Until lately, however, the absence of a Leydig cell-exclusive Cre driver mouse line made this strategy impractical. The recent development of the *Insl3*^iCre^ mouse line (9) now provides the necessary specificity, making targeted Leydig cell ablation using the iDTR system fully feasible.

In this study, we employed the iDTR/DT system together with our Leydig cell-exclusive *Insl3*^iCre^ line to ablate all differentiated Leydig cells. Our findings demonstrate that Leydig cells can be rapidly eliminated in adult mice through DT administration, after which a new generation of functional Leydig cells emerges from resident SLCs. The acute loss of functional Leydig cells produced a transient atrophy of androgen-dependent secondary sex organs, reflecting the temporary but sharp decline in testosterone production. Additionally, our results support a role for macrophages both in clearing ablated Leydig cells and in facilitating the establishment of the newly differentiated Leydig cell population.

## Materials and methods

### Animals

Mice were maintained in specific pathogen-free conditions, a temperature-controlled environment (23-25 °C), a 12:12-h light–dark cycle and had unlimited access to food and water. All experiments were conducted according to the Canadian Council for Animal Care and were approved by the Animal Care and Ethics Committee of Université Laval (protocol #2022-1047, CHU-22-1047). DT was used in containment level 2 according to the Canadian Biosafety Standard and approved by the Public Health Agency of Canada (permit #C-22-1040-01-JJT04). B6C3H;C57BL/6N-*Insl3*^em2(iCre)Trejj^ mice, commonly as *Insl3*^iCre^, expressing the improved Cre recombinase (iCre) exclusively in Leydig cells were described previously (9). The B6.Cg-*Gt(ROSA)26Sor^tm9(CAG-tdTomato)Hze^*/J mouse line (37) that contains a CAG-LoxP-STOP-LoxP-TdTomato-WPRE cassette inserted into the *Rosa26* locus (*Rosa26*^LSL-TdTomato^ from here on) was obtained from The Jackson Laboratories (strain #007909; Bar Harbor, ME, USA; RRID:IMSR_JAX:007909). The C57BL/6-*Gt(ROSA)26Sor^tm1(HBEGF)Awai^*/J mouse line (38) was obtained from The Jackson Laboratories (strain #:007900, RRID:IMSR_JAX:007900). In these mice, a STOP cassette flanked by LoxP sites followed by the gene encoding the simian iDTR was inserted into the *Rosa26* locus (*Rosa26*^LSL-iDTR^). When *Rosa26*^LSL-TdTomato/LSL-iDTR^ mice are crossed with mice expressing a Cre recombinase, the STOP cassette is removed resulting in robust TdTomato fluorescence and iDTR expression in Cre-expressing cells. Mice were genotyped using genomic DNA from ear punches as previously described (9) using the primers and conditions listed in Table 1. PCRs were performed using 2X Taq FroggaMix (FroggaBio, Concord, Ontario, Canada) according to manufacturer's instructions. Adult (50 days; P50) *Insl3*^iCre^;*Rosa26*^TdTomato/iDTR^ male mice were administered either saline (Saline control) or DT (40 ng/g body weight) (Sigma-Aldrich Canada, Oakville, ON, Canada; Cat# D0564-1MG) intraperitoneally (IP) for 3 consecutive days. Adult (50 days; P50) *Insl3*^iCre^;*Rosa26*^TdTomato^ males lacking iDTR were also administered DT according to the same protocol and used as a control (DT control). Between 5 and 12 mice were used per timepoint and per treatment.

**Table 1:** Oligonucleotides used in this study

Application	Description	Sequences	PCR conditions
Genotyping (PCR)	*iCre*	(F): 5′-TCTGATGAAGTCAGGAAGAACC-3′ (R): 5′-GAGATGTCCTTCACTCTGATTC-3′	Initial denaturation 95 °C, 3 minutes; 35 cycles: 95 °C, 30 second/60 °C, 30 second/72 °C, 30 second; final elongation 72 °C, 5 minutes
	*Insl3* wild-type (WT)	(F): 5′-GACATGTGGGCTTCACCCTT-3′ (R): 5′-ACTCCAGTCTTTGCTATCAGGAGG-3′	Initial denaturation 95 °C, 3 minutes; 35 cycles: 95 °C, 30 second/62 °C, 30 second/72 °C, 30 second; final elongation 72 °C, 5 minutes

### Blood, organ collection, and processing

At 4, 7, 14, 35, and 65 days postsaline or DT administration, *Insl3*^iCre/+^;*Rosa26*^TdTomato/iDTR^ or *Insl3*^iCre/+^;*Rosa26*^TdTomato/+^ males were weighed before being euthanized. While under terminal anesthesia, blood was collected by cardiac puncture, mixed with EDTA in 1.5 mL tubes, and centrifuged at 3000 rpm for 15 minutes. The supernatant was then harvested and kept at −80 °C until testosterone quantification. Reproductive organs, including the testis, epididymis, seminal vesicles, were collected and weighted. For each animal, 1 testis was snap-frozen in dry ice and kept at −80 °C for intratesticular testosterone quantification. The other testis was fixed, embedded in optimal cutting temperature compound (Tissue-Tek, Sakura, VWR International, Ville Mont-Royal, Quebec, Canada), and snap-frozen by immersing the mold into liquid nitrogen-chilled isopentane as previously described (9). Tissues were left for at least 1 hour at −20 °C before being cut using a cryostat (Leica CM3050S).

### Direct fluorescence and immunofluorescence

Six-micrometer sections of optimal cutting temperature-embedded samples were prepared using a cryostat (Leica CM3050S) and mounted on Superfrost Plus slides (Fisher Scientific, Ottawa, Ontario, Canada). For direct visualization of TdTomato fluorescence, sections were rehydrated in PBS, and counterstained with VECTASHIELD Antifade Mounting Medium with 4',6-diamidino-2-phenylindole (DAPI) (MJS BioLynx, Brockville, Ontario, Canada) as described in Ref. (9). Images were captured using an epifluorescence microscope (Nikon Eclipse 80i, Nikon Canada, Mississauga, Ontario, Canada) or with a Zeiss Axio Imager M2 epifluorescence microscope, Axiocam 705 camera (Zeiss Laboratories, Germany). For immunofluorescence, sections were rehydrated in PBS and subjected to antigen retrieval using 1 of 2 methods depending on the primary antibodies used: incubation in sodium citrate buffer, pH 6.0, at 80 °C for 15 minutes using a Decloaking Chamber NxGen (Biocare Medical, Pacheco, CA, USA), or treatment with 1% sodium dodecyl sulfate for 5 minutes at room temperature. Following antigen retrieval, sections were permeabilized with 0.3% Triton X-100 for 15 minutes, blocked in PBS + 10% donkey serum for 1 hour at room temperature, and incubated overnight at 4 °C with either an anti-CYP17A1 goat antiserum (1:200; Santa Cruz Biotechnology, sc-46081; RRID:AB_2088659), an anti-alpha-smooth muscle actin mouse monoclonal antibody (anti-αSMA; 1:4000; Sigma-Aldrich Cat# A5228, RRID:AB_262054), an anti-alpha-smooth muscle actin rabbit polyclonal antibody (anti-αSMA; 1:200; Abcam Cat# ab5694, RRID:AB_2223021), an anti-COUP-TFII/NR2F2 mouse monoclonal antibody (anti-αCOUP-TFII/NR2F2; 1:100; clone H7147; R&D Systems; Cat# PP-H7147-00, RRID:AB_2155627), an anti-active Caspase-3 rabbit polyclonal antibody (1:200; R&D Systems; Cat# AF835, RRID:AB2243952), or an anti-F4-80 rat monoclonal antibody (1:100; clone BM8; Thermo Fisher Scientific; Cat# 14-4801-85, RRID:AB_467559). The next morning, the slides were rinsed 3× in PBS and incubated with a secondary antibody for 1 hour at room temperature: for CYP17A1, a donkey antigoat IgG secondary antibody coupled to Alexa Fluor 488 (1:200; Thermo Fisher Scientific Cat# A-11055; RRID:AB_2534102), for αSMA, a goat antimouse IgG secondary antibody coupled to Alexa Fluor 488 (1:200; Thermo Fisher Scientific Cat# A-11001; RRID:AB_2534069) or a goat antirabbit IgG (H + L) coupled to DyLight 405 (1:200; Jackson ImmunoResearch, West Grove, PA, USA; Cat# 111-475-045, RRID:AB_2338038), for COUP-TFII/NR2F2 a goat antimouse IgG2a coupled to Alexa Fluor 647 (1:200; Invitrogen Cat# A-21241; RRID:AB_2535810), for active Caspase-3 a biotinylated donkey antirabbit IgG (H + L) (1:200; Jackson ImmunoResearch, West Grove, PA, USA; Cat# 711-065-152, RRID:AB_2340593), and for F4-80 a biotinylated donkey antirat IgG (1:200; Jackson ImmunoResearch, West Grove, PA, USA; Cat# 712-065-153, RRID:AB_2315779). For active Caspase-3 and F4-80, incubation with the biotinylated secondary antibodies was followed by streptavidin coupled to Alexa Fluor 647 (1:200; Jackson ImmunoResearch; Cat# 016-600-084, RRID:AB_2341101). The slides were then rinsed 3× in PBS and counterstained with DAPI as described previously (9). Omission of primary antisera was used as negative control.

### Intratesticular and plasma testosterone quantification

For intratesticular testosterone quantification, testis fragments were homogenized using a Polytron homogenizer (VWR 200 Homogenizer; VWR International) in 500 μL of cold PBS. The resulting solution was then centrifuged at 3000 rpm for 5 minutes at 4 °C and the supernatant containing the testosterone was kept at −80 °C until quantification. Testosterone levels in blood plasma (1:20 or 1:50 dilution) and in testicular homogenates (1:100 dilution) were determined by ELISA (Cayman Chemical, Ann Arbor, MI, USA; Cat# 582701, RRID:AB_2895148) following the manufacturer's recommendations as described previously (39).

### Statistical analyses

Data are presented as the mean ± SEM; the number of replicates is indicated on each figure. The nonparametric Kruskal–Wallis test for multiple comparisons (Mann–Whitney U with Bonferroni correction) was used to analyze weight variation over time within a single group of mice, as well as to assess weight differences between each group at a given time. This statistical test was also used to compare testosterone levels over time within the same group of mice. To compare testosterone levels in the DT group with those in the control group (DT control) at each time point, an unpaired *t*-test was performed using GraphPad Prism 9 graphing software (GraphPad Software, San Diego, CA, USA). The results were considered statistically significant when the *P* value was less than .05.

## Results

### Specific elimination of functional Leydig cells in adult mice

SLCs have been shown to persist in the adult testis, and their ability to differentiate into a new population of functional ALCs has been characterized primarily in rat models treated with the cytotoxic alkylating compound EDS. In contrast, EDS does not effectively ablate Leydig cells in mice, limiting its usefulness in that species. A DT-mediated Leydig cell ablation approach was therefore developed by crossing the *Insl3*^iCre^ mouse line that constitutively expresses the Cre recombinase exclusively in Leydig cells (9) with the *Rosa26*^LSL-TdTomato/LSL-iDTR^ line. In the resulting male offspring (*Insl3*^iCre^;*Rosa26*^TdTomato/iDTR^), all Leydig cells express the red fluorescent TdTomato protein and the diphtheria toxin receptor (iDTR). Experiments were first conducted to identify the optimal DT administration protocol. As shown in Fig. 1A, IP of DT at 40 ng/g body weight (bw) on 2 consecutive days was markedly more effective at eliminating red-labeled Leydig cells than a 10 ng/g bw dose when evaluated 4 days post-DT treatment. Increasing the regimen to 40 ng/g bw for 3 consecutive days removed the vast majority of Leydig cells (Fig. 1B). Neither DT administration in control mice lacking iDTR (*Insl3*^iCre^;*Rosa26*^TdTomato/+^) nor saline injection in iDTR-expressing mice (*Insl3*^iCre^;*Rosa26*^TdTomato/iDTR^) affected animal health or Leydig cell numbers (Fig. 1C), confirming both the safety and specificity of the model.

**Figure 1:**
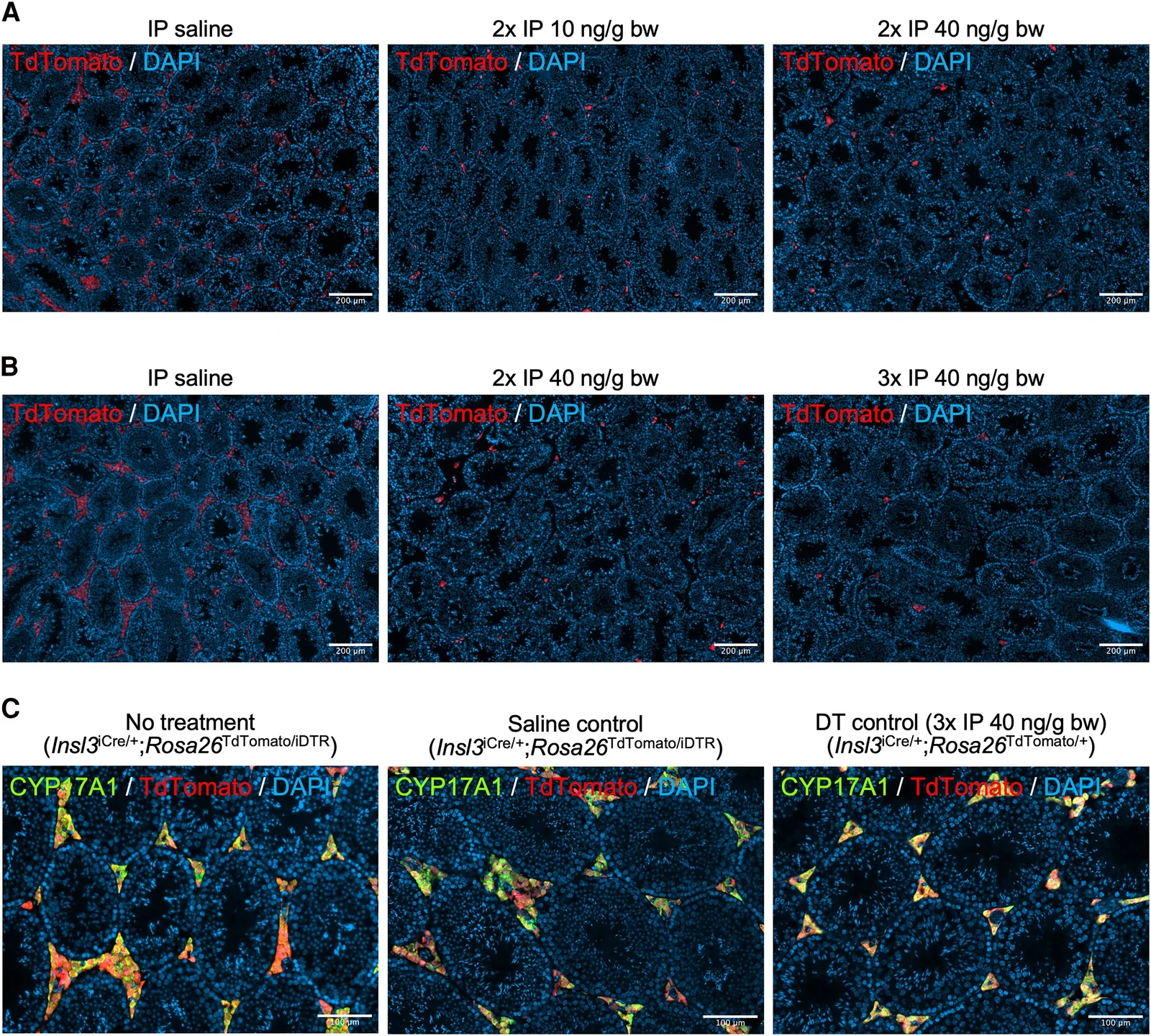
Optimization of DT-mediated ALC ablation. Testes from P50 *Insl3*^iCre/+^;*Rosa26*^TdTomato/iDTR^ mice were analyzed on cryosections by direct TdTomato fluorescence to assess Leydig cell depletion following administration of saline or different DT regimens. (A) Comparison of 2 DT doses (10 and 40 ng/g body weight, bw). (B) Comparison of 2 vs 3 intraperitoneal (IP) DT injections. (C) Immunofluorescence staining for CYP17A1 was performed to evaluate Leydig cell abundance in the indicated control and DT-treated groups. Nuclei were counterstained with DAPI. Scale bars = 100 µm.

### DT-mediated Leydig cell ablation induces apoptosis

To determine whether DT-mediated Leydig cell depletion involved apoptosis, immunofluorescence for activated Caspase 3 was performed at 4 days post-DT, when Leydig cell loss was pronounced. Activated Caspase 3-positive cells were detected within the testicular interstitium and colocalized with TdTomato-positive Leydig cells or TdTomato-positive cellular remnants (Fig. 2). In contrast, activated Caspase 3-positive cells were not detected at 65 days post-DT, when the Leydig cell population had been fully restored (Fig. 2). Together, these findings indicate that apoptosis is associated with the acute phase of DT-mediated ablation of the targeted Leydig cell population.

**Figure 2:**
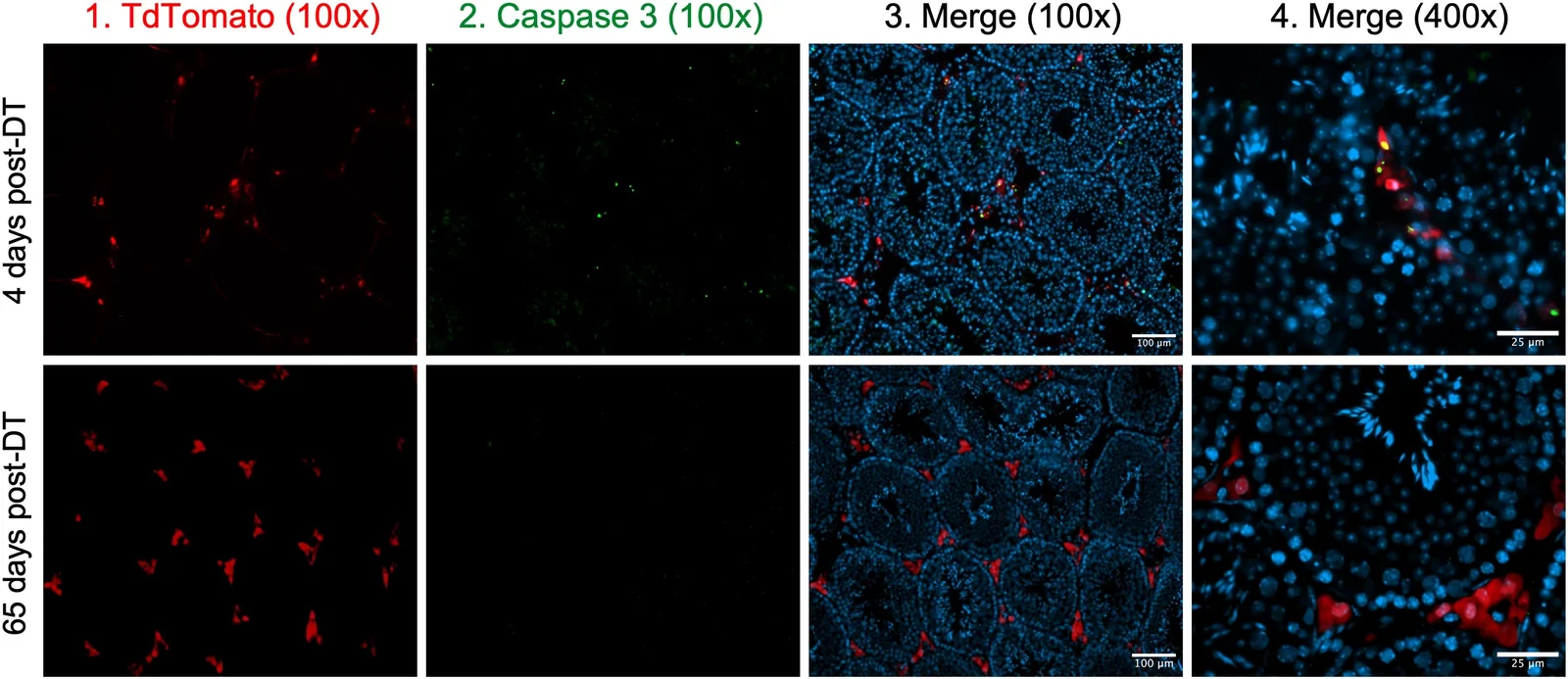
DT-mediated Leydig cell ablation is associated with apoptosis. Cryosections of testes from P50 *Insl3*^iCre/+^;*Rosa26*^TdTomato/iDTR^ mice collected 4 and 65 days following DT administration were analyzed by direct detection of TdTomato fluorescence and immunofluorescence for activated Caspase 3. Individual TdTomato and activated Caspase 3 channels and merged images are shown. The higher-magnification image illustrates colocalization of activated Caspase 3 with TdTomato-positive Leydig cells/cellular remnants at 4 days post-DT. Scale bars are indicated on the images.

### Appearance of a new generation of functional Leydig cells

To assess whether SLCs persist in the adult mouse testis and can regenerate a new population of functional ALCs, adult *Insl3*^iCre^;*Rosa26*^TdTomato/iDTR^ males were treated with DT at 40 ng/g body weight for 3 consecutive days and sacrificed at various time points (Fig. 3A). As shown in Fig. 3B, by 4 days post-DT, nearly all Leydig cells, identified by red fluorescence and CYP17A1 expression, were eliminated. By 7 days post-DT, Leydig cell ablation was essentially complete, leaving the interstitial space largely devoid of Leydig cells. Newly emerging TdTomato-positive cells were observed, some of which had not yet acquired CYP17A1 expression (white arrows in Fig. 3B), consistent with an early stage of Leydig cell differentiation. A few TdTomato-positive cells had already acquired CYP17A1 expression (yellow arrowheads in Fig. 3B), indicating progression toward a steroidogenic Leydig cell phenotype. By 14 days post-DT, newly formed TdTomato-positive/CYP17A1-positive Leydig cells were increasingly evident, with some extending away from the peritubular region into the interstitial compartment, while differentiation remained ongoing. By 35 days post-DT, the number of regenerated Leydig cells had increased substantially, and these cells expressed CYP17A1 as they repopulated the interstitial space. By 65 days post-DT, a fully reconstituted population of functional Leydig cells occupied the interstitium, representing a completely new generation of ALCs (Fig. 3B).

**Figure 3:**
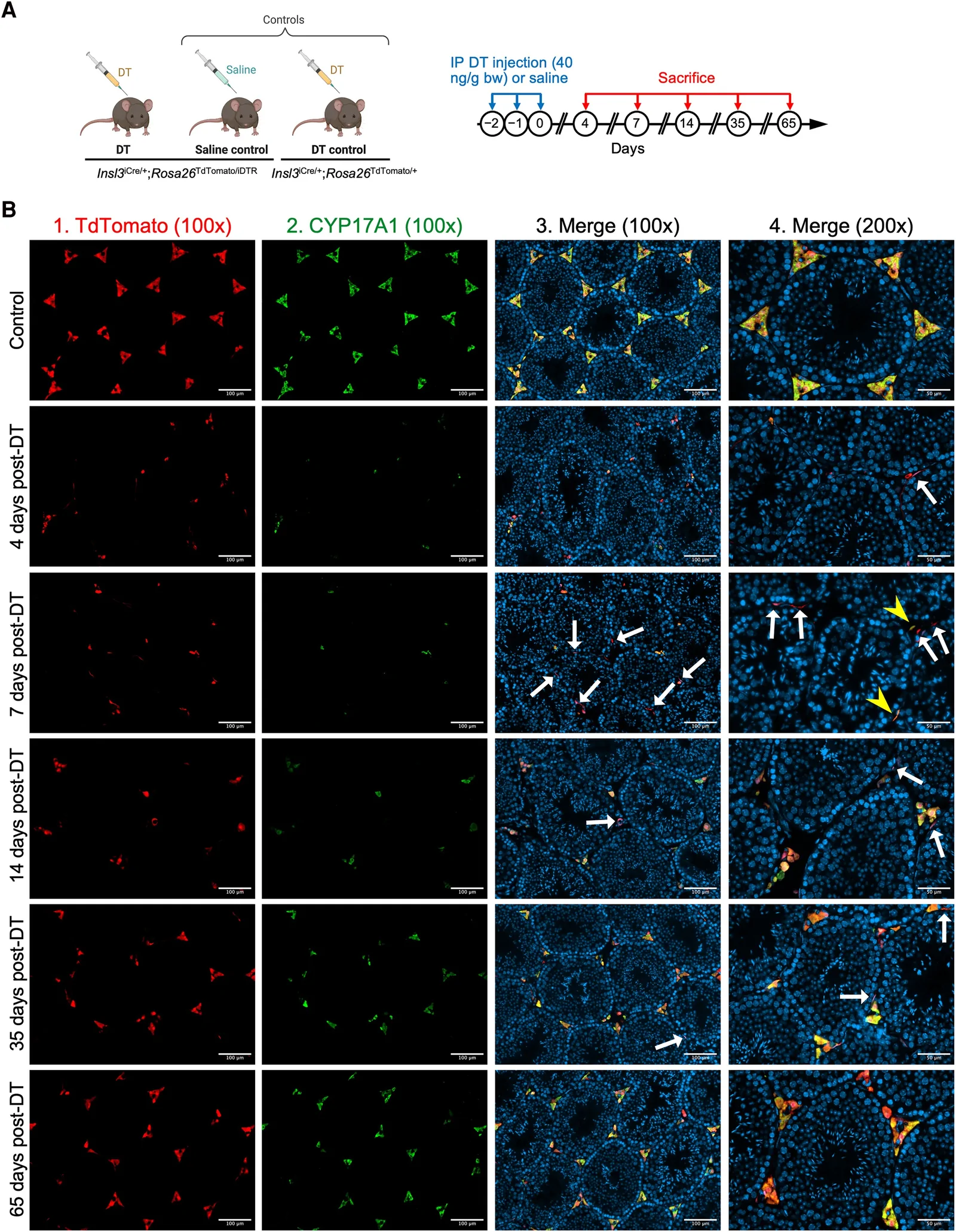
DT-mediated ablation of ALCs and regeneration of a new Leydig cell population from resident SLCs. (A) Experimental design used to selectively ablate ALCs in *Insl3*^iCre/+^;*Rosa26*^TdTomato^/^iDTR^ mice by DT administration. (B) Cryosections of testes collected from *Insl3*^iCre/+^;*Rosa26*^TdTomato^/^iDTR^ and control *Insl3*^iCre/+^;*Rosa26*^TdTomato/+^ P50 mice at 4, 7, 14, 35, and 65 days post-DT following saline or DT treatment were analyzed for Leydig cell abundance by direct detection of TdTomato fluorescence. Immunofluorescence staining for CYP17A1 was performed to identify differentiated Leydig cells. Nuclei were counterstained with DAPI. Individual fluorescence channels and merged images are shown at the indicated magnifications. White arrows indicate newly emerging TdTomato-positive/CYP17A1-negative cells, whereas yellow arrowheads indicate differentiating TdTomato-positive/CYP17A1-positive Leydig cells. Scale bars are indicated on the images.

### COUP-TFII-positive peritubular precursors persist after Leydig cell ablation

To further characterize the precursor population that survives DT-mediated Leydig cell ablation, immunofluorescence for COUP-TFII/NR2F2 was performed at 4 days post-DT. At this time point, *Insl3*-positive Leydig cells were nearly completely depleted, while newly emerging TdTomato-positive cells were beginning to appear (Fig. 3B). COUP-TFII-positive cells were detected immediately adjacent to αSMA-positive peritubular myoid cells (PMCs) surrounding seminiferous tubules (white arrows in Fig. 4). These cells were αSMA-negative, CYP17A1-negative, and TdTomato-negative, distinguishing them from αSMA-positive PMCs (white arrowheads in Fig. 4) and TdTomato-positive Leydig-lineage cells. Notably, some COUP-TFII-positive cells at the peritubular surface had begun to acquire TdTomato fluorescence (yellow arrows in Fig. 4), consistent with activation of *Insl3* and entry into the Leydig cell lineage. Together with the subsequent appearance of TdTomato-positive Leydig cells predominantly within the peritubular compartment, these findings identify a COUP-TFII-positive, αSMA-negative, CYP17A1-negative, and *Insl3*/TdTomato-negative population as a strong candidate source of regenerating ALCs and indicate that these cells share molecular characteristics with the steroidogenic progenitor populations described during testicular development.

**Figure 4:**
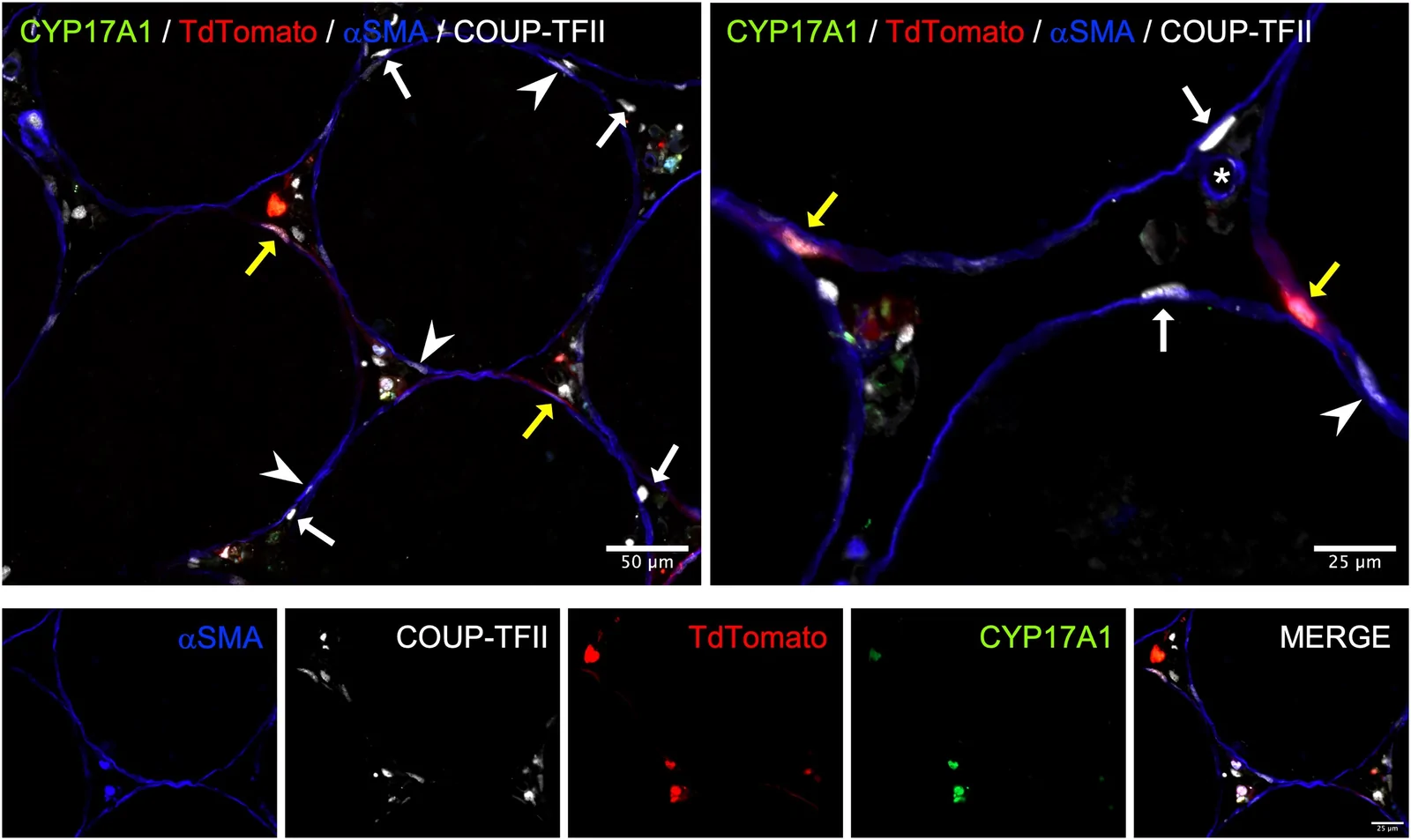
Identification of COUP-TFII-positive candidate Leydig precursor cells following DT-mediated Leydig cell ablation. Cryosections of testes from P50 *Insl3*^iCre/+^;*Rosa26*^TdTomato/iDTR^ mice collected 4 days post-DT were analyzed by immunofluorescence for COUP-TFII/NR2F2, αSMA, and CYP17A1 together with direct detection of TdTomato fluorescence. COUP-TFII-positive cells located immediately adjacent to the αSMA-positive PMC layer and negative for αSMA, CYP17A1, and TdTomato are indicated by white arrows. COUP-TFII was also detected in αSMA-positive PMCs (white arrowheads) and in TdTomato-positive/CYP17A1-positive Leydig cells. COUP-TFII-positive cells located at the peritubular surface that were also positive for TdTomato are indicated by yellow arrows. Individual fluorescence channels and merged images are shown. Scale bars are indicated on the images.

In mice, ALCs begin to differentiate during postnatal development (P5-P7) from progenitor cells located adjacent to PMCs surrounding seminiferous tubules and, to a lesser extent, perivascular myoid cells surrounding blood vessels (9). To determine whether the newly formed Leydig cells that generated following DT-mediated ablation arise from the same cellular source, immunofluorescence staining for αSMA, a marker of peritubular and perivascular myoid cells, was performed on testis sections 14 days post-DT administration. As shown in Fig. 5A, elongated red-labeled αSMA-negative cells were observed adjacent to αSMA-positive myoid cells surrounding both blood vessels (Fig. 5A, white arrow) and seminiferous tubules (Fig. 5A, yellow arrowhead). Immunofluorescence analysis of VCAM1, a marker of testicular mesenchymal progenitor cells and differentiated ALCs (40), revealed that these newly committed Leydig cells (labeled red) also express VCAM1 (Fig. 5B, left), as do differentiated, functional ALCs (Fig. 5B, right). Collectively, these findings indicate that adult SLCs originate from VCAM1^+^ mesenchymal progenitor cells located adjacent to peritubular and perivascular myoid cells.

**Figure 5:**
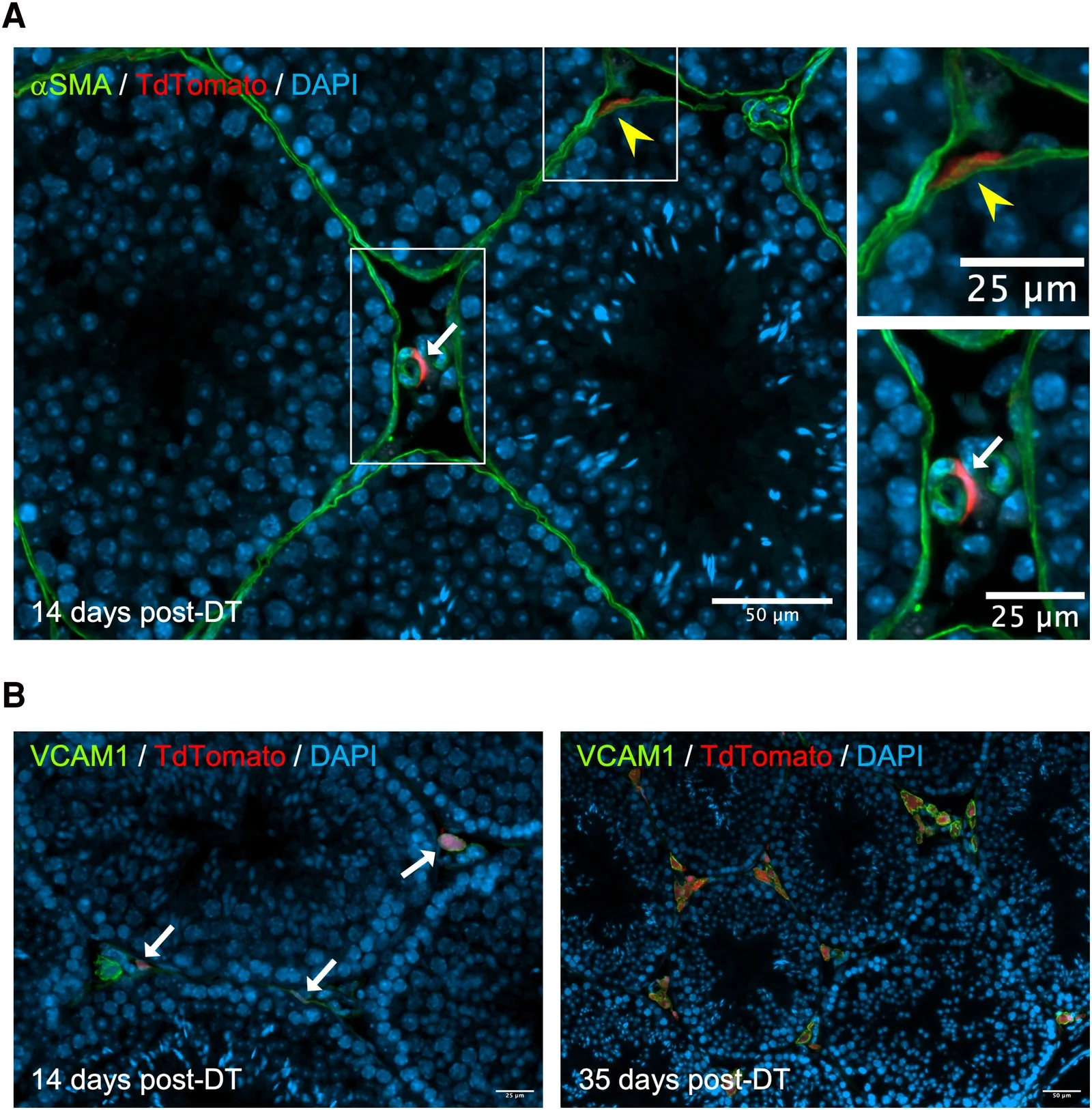
Regeneration of Leydig cells from peritubular and perivascular progenitor cells following DT-mediated ablation. Cryosections of testes from P50 *Insl3*^iCre/+^;*Rosa26*^TdTomato^/^iDTR^ mice were collected 14 and 35 days post-DT administration. Developing and regenerated Leydig cells were identified by direct detection of TdTomato fluorescence. Nuclei were counterstained with DAPI. (A) Immunofluorescence staining for αSMA, a marker of peritubular and perivascular myoid cells, revealed newly committed Leydig cells adjacent to αSMA-positive myoid cells surrounding blood vessels (white arrow) and seminiferous tubules (yellow arrowhead). (B) Immunofluorescence staining for VCAM1, a marker of testicular mesenchymal progenitor cells and ALCs. Newly formed Leydig cells at day 14 (left) and differentiated, functional ALCs at day 35 (right) post-DT expressed VCAM1. Scale bars are indicated on each image.

### Physiological impact of Leydig cell ablation

We next assessed the impact of Leydig cell ablation on testosterone production. Four days post-DT administration, when Leydig cells were effectively depleted, intratesticular (Fig. 6A) and circulating (Fig. 6B) testosterone levels were nearly undetectable. Testosterone concentrations remained low at 7 days post-DT but began to increase by day 14, coinciding with the emergence of newly differentiated CYP17A1-positive Leydig cells. By 35 and 65 days post-DT, both intratesticular and plasma testosterone levels had returned to control values (Fig. 6). Consistent with the decline in testosterone, the epididymides and seminal vesicles, both androgen-dependent organs, showed marked reductions in size (Fig. 7) and weight (Fig. 8) at 4 and 7 days post-DT. These parameters returned to normal as regenerated Leydig cells resumed testosterone production, beginning at 14 days post-DT. In contrast, neither body weight nor testis weight was significantly affected during DT-mediated Leydig cell ablation or throughout the subsequent regeneration of functional Leydig cells (Fig. 8).

**Figure 6:**
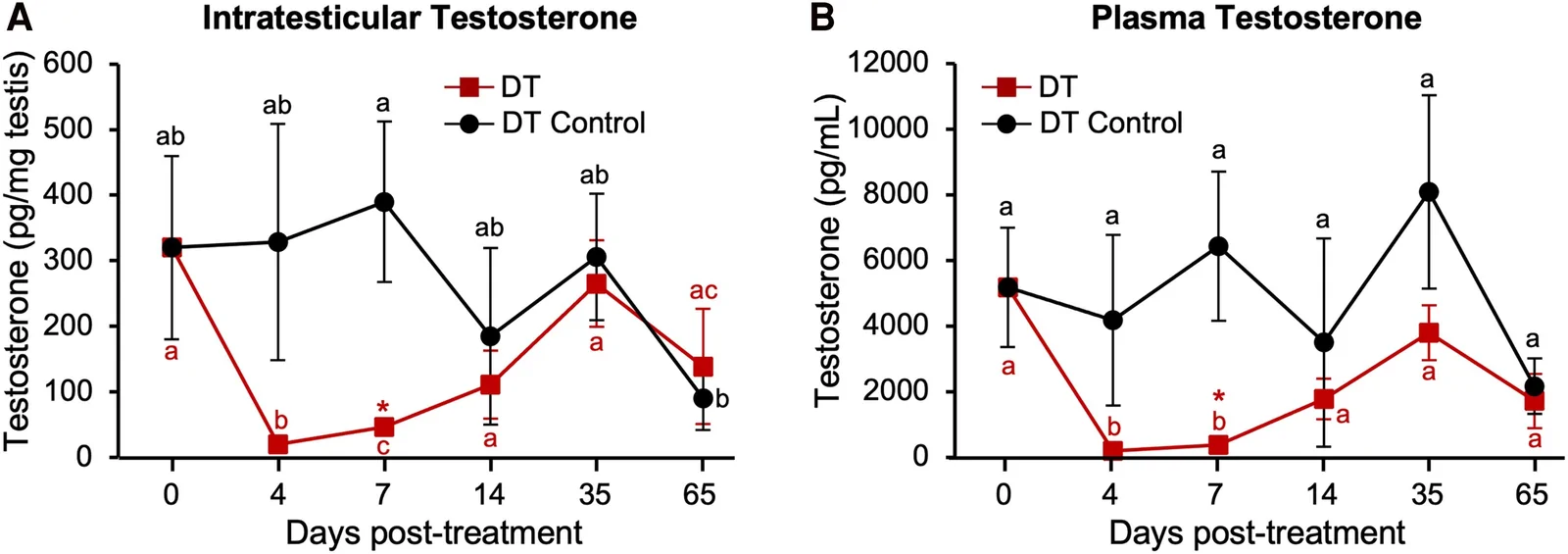
Transient suppression of intratesticular and circulating testosterone levels following DT-mediated Leydig cell ablation. Intratesticular (A) and plasma (B) testosterone concentrations were measured at the indicated time points following DT administration in P50 male *Insl3*^iCre/+^;*Rosa26*^TdTomato^/^iDTR^ mice (DT; squares) and control *Insl3*^iCre/+^;*Rosa26*^TdTomato/+^ littermates lacking the DT receptor (DT control; circles). Intratesticular testosterone levels are expressed as pg/mg testis, and plasma testosterone levels as pg/mL. Data are presented as mean ± SEM (*n* = 5-10 mice per group). Asterisks (*) denote significant differences between DT-treated and control groups at a given time point (*P* < .05; unpaired *t*-test). Different letters indicate significant differences among time points within the same experimental group (*P* < .05; Kruskal–Wallis ANOVA).

**Figure 7:**
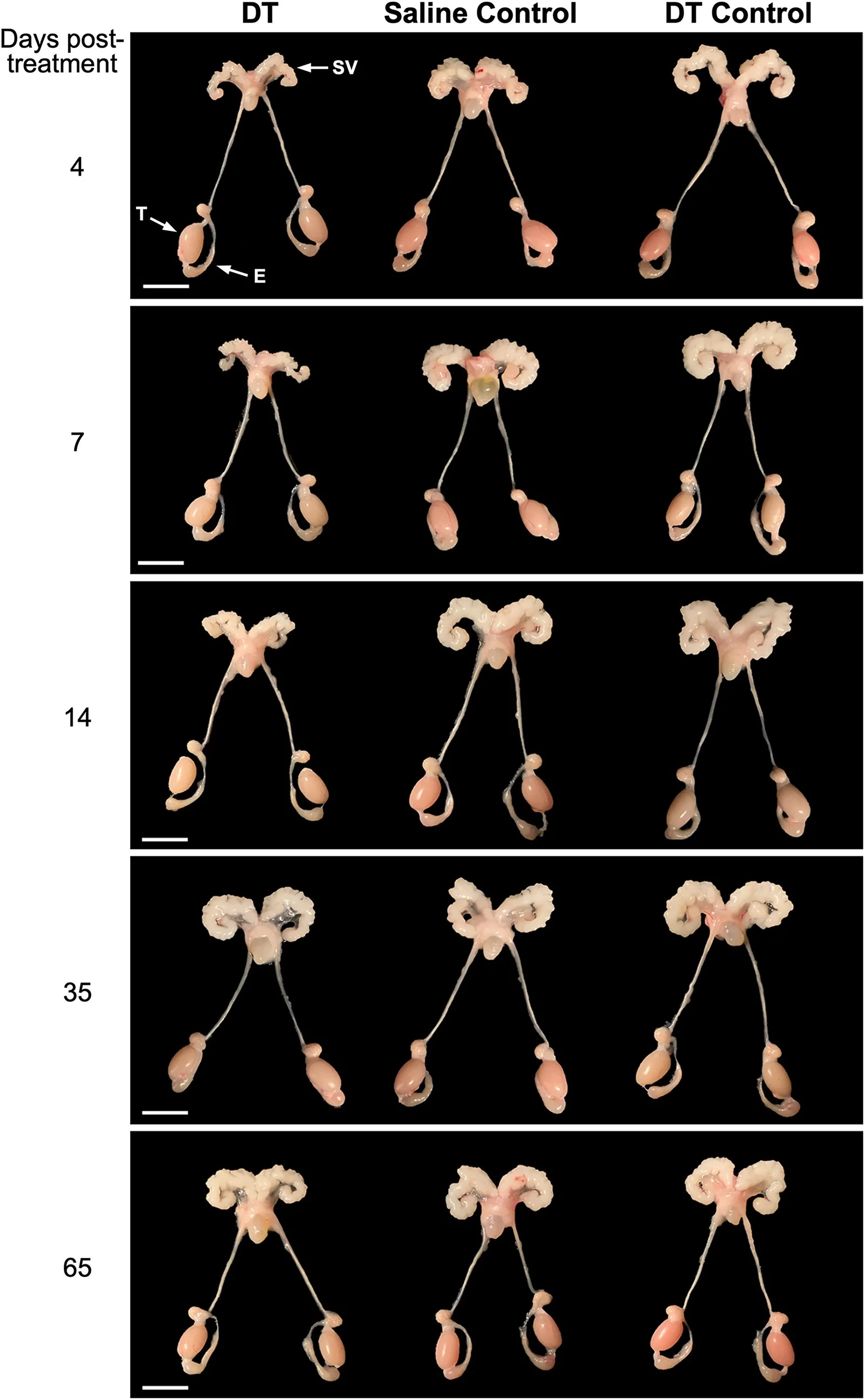
Transient atrophy of androgen-dependent organs following DT-mediated Leydig cell ablation. Representative gross morphology of the testes (T), seminal vesicles (SV), and epididymides (E) from P50 male *Insl3*^iCre/+^;*Rosa26*^TdTomato^/^iDTR^ and control *Insl3*^iCre/+^;*Rosa26*^TdTomato/+^ mice at the indicated time points following saline or DT treatment. Scale bar = 1 cm.

**Figure 8:**
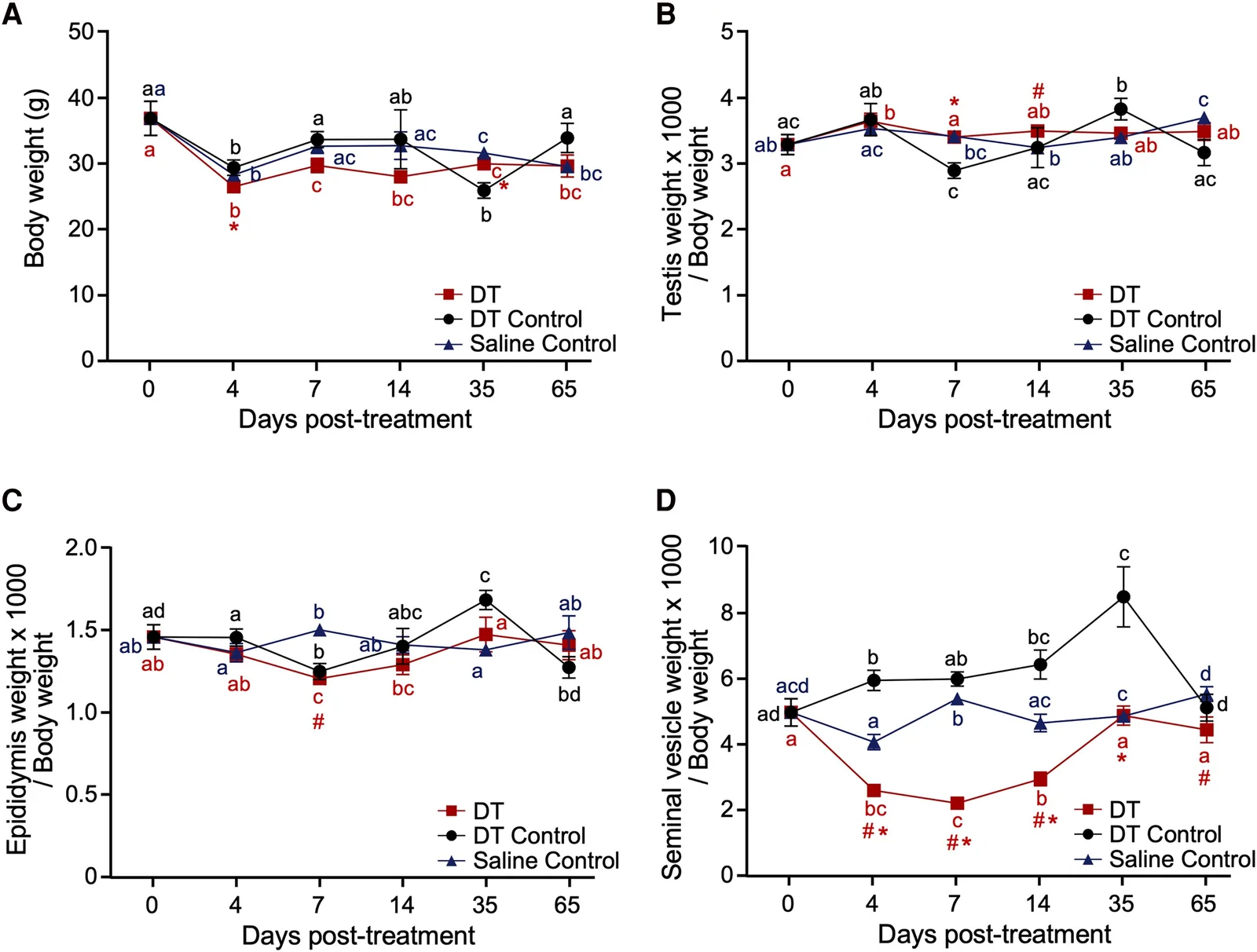
Transient reduction in the weight of androgen-dependent reproductive organs following DT-mediated Leydig cell ablation. Body weight (A) and organ-to-body weight ratios for testes (B), epididymides (C), and seminal vesicles (D) were determined at the indicated time points following saline or DT treatment of P50 male *Insl3*^iCre/+^;*Rosa26*^TdTomato^/^iDTR^ mice (DT, squares; saline control, triangles) and *Insl3*^iCre/+^;*Rosa26*^TdTomato/+^ control mice lacking the DT receptor (DT control, circles). Data are presented as mean ± SEM (*n* = 5-12 mice per group). Asterisks (*) indicate significant differences between the DT and DT control groups at a given time point (*P* < .05), whereas hashtags (#) indicate significant differences between the DT and saline control groups (*P* < .05) (unpaired *t*-test). Different letters denote significant differences among time points within the same experimental group (*P* < .05; Kruskal–Wallis ANOVA).

### Involvement of macrophages in Leydig cell ablation and regeneration

Testicular macrophages and Leydig cells are known to maintain a close functional relationship (reviewed in Ref. (41)). To investigate whether macrophages contribute to the clearance of apoptotic Leydig cells following DT treatment, immunofluorescence staining for F4-80, a marker of both interstitial and peritubular testicular macrophages, was performed. In control testes, resident macrophages were present at low density throughout the interstitium (Fig. 9). Consistent with the presence of activated Caspase-3-positive apoptotic Leydig cells at day 4 post-DT (Fig. 2) when most functional Leydig cells had been eliminated (Fig. 3), F4-80-positive macrophages accumulated within the interstitial compartment at 4 and 7 days post-DT (Fig. 9). Notably, remnants of dead Leydig cells were observed within macrophages (white arrowheads in Fig. 9), consistent with phagocytic clearance of apoptotic Leydig cells. At 14 and 35 days post-DT, a newly emerging population of Leydig cells was detected in close association with macrophages (Fig. 9). By 65 days post-DT, when Leydig cell regeneration was complete, macrophage numbers had returned to baseline levels comparable to those observed in control testes (Fig. 9). Together, these findings demonstrate that testicular macrophages participate in the phagocytosis and clearance of apoptotic Leydig cells following DT-mediated ablation.

**Figure 9:**
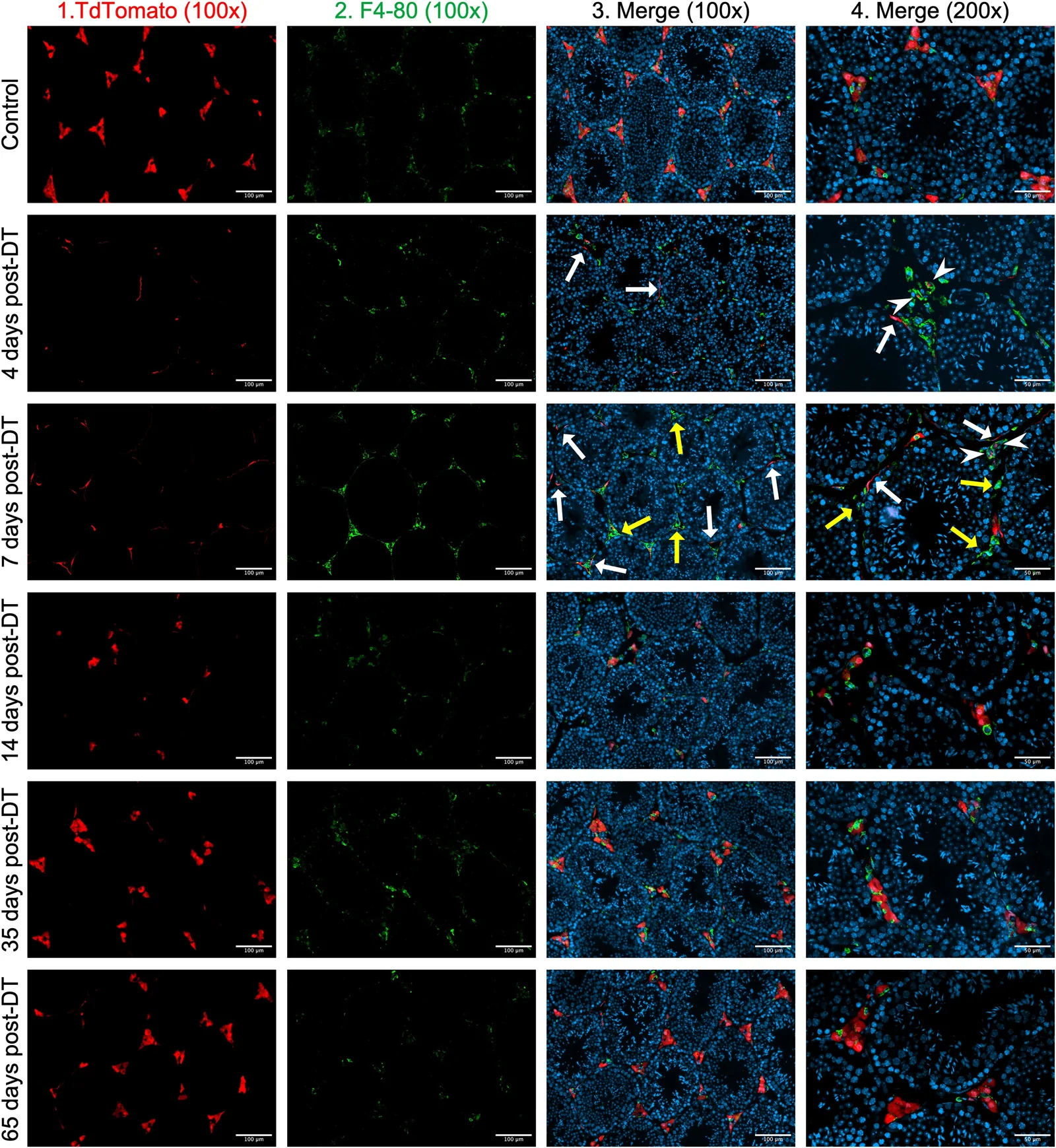
Increased macrophage recruitment during Leydig cell ablation and regeneration. Cryosections of testes from P50 *Insl3*^iCre/+^;*Rosa26*^TdTomato/+^ control mice and *Insl3*^iCre/+^;*Rosa26*^TdTomato/iDTR^ mice collected 4, 7, 14, 35, and 65 days post-DT administration were analyzed by direct detection of TdTomato fluorescence (red) to visualize Leydig cells. Immunofluorescence staining for F4-80 (green) was performed to identify testicular macrophages. White arrowheads indicate remnants of TdTomato-positive Leydig cells enclosed within F4-80-positive macrophages (green). Nuclei were counterstained with DAPI (blue). Individual fluorescence channels and merged images are shown at the indicated magnifications. Scale bars are indicated on each image.

## Discussion

Despite decades of work demonstrating Leydig cell regeneration after injury, the cellular origin of regenerated Leydig cells and the microenvironment supporting this process remain incompletely defined. Progress has been hampered by the lack of mouse models that permit efficient and specific ablation of the Leydig cell lineage while allowing long-term regenerative follow-up. In the present study, we established an *Insl3*^iCre^*;Rosa26*^TdTomato/iDTR^ model that enables selective elimination of all committed Leydig-lineage cells and permanent fluorescent labeling of regenerated cells. Using this approach, we show that complete depletion of *Insl3*-expressing Leydig cells is followed by regeneration of a fully functional Leydig cell population, providing direct evidence for the existence of regenerative Leydig precursors in the adult mouse testis (Fig. 10).

**Figure 10:**
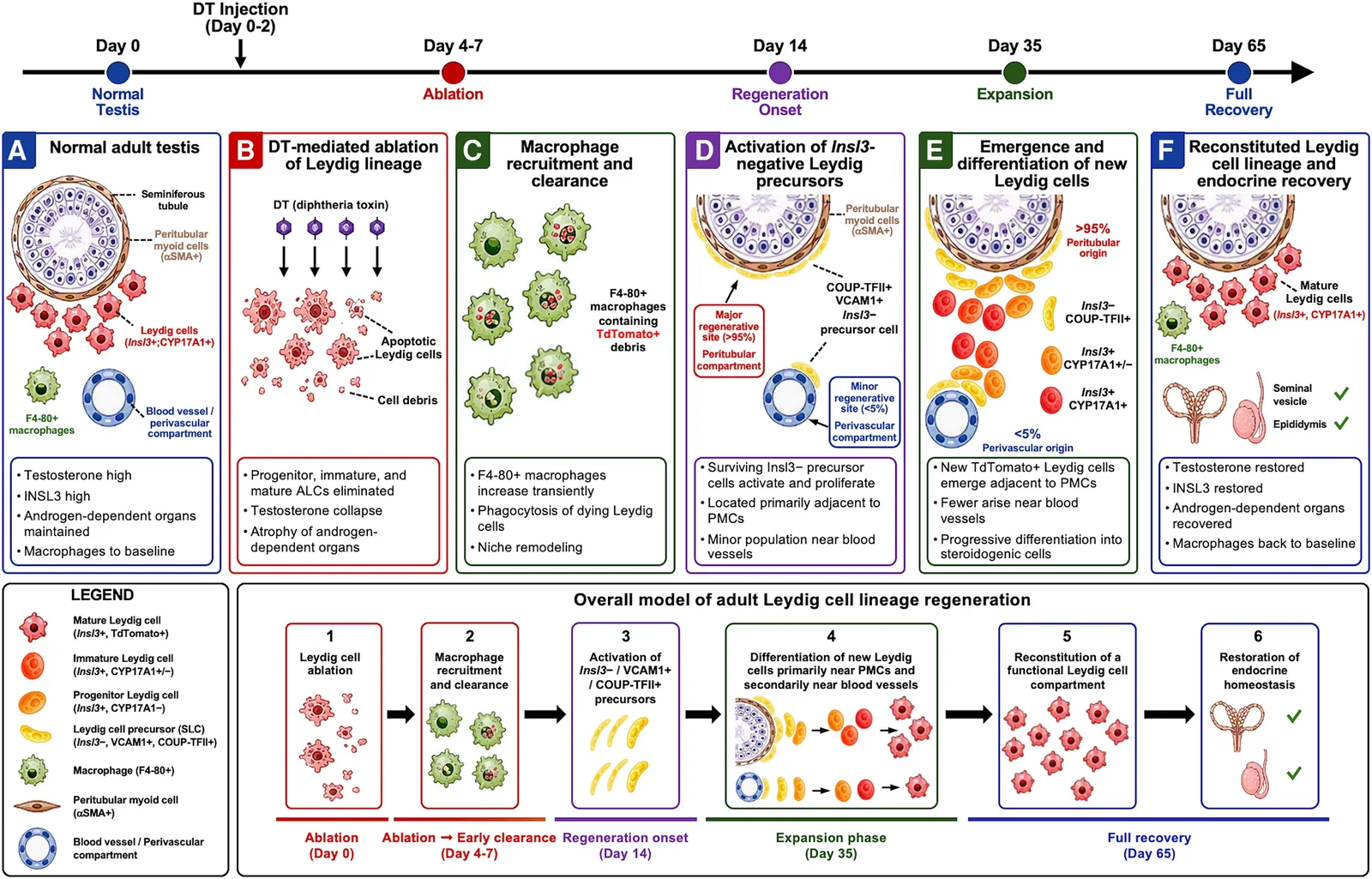
Proposed cellular dynamics underlying Leydig cell regeneration after complete lineage ablation. (A) Under normal conditions, *Insl3*-positive/CYP17A1-positive ALCs populate the interstitial compartment and maintain testosterone and INSL3 production. (B) DT administration eliminates *Insl3*-positive progenitor, immature, and mature ALCs, resulting in loss of testosterone production and atrophy of androgen-dependent organs. (C) During the acute ablation phase, F4-80-positive macrophages transiently accumulate and clear apoptotic Leydig cells and TdTomato-positive cellular debris. (D) Surviving *Insl3*-negative/COUP-TFII-positive/VCAM1-positive Leydig precursor cells located primarily adjacent to PMCs and, to a lesser extent, perivascular are activated. (E) Newly committed *Insl3*-positive/TdTomato-positive Leydig cells emerge and progressively acquire CYP17A1 expression as the regenerated population expands. (F) Reconstitution of the mature *Insl3*-positive/CYP17A1-positive Leydig cell population restores testosterone and INSL3 production, androgen-dependent organ function, and endocrine homeostasis, while macrophage abundance returns to baseline.

### An efficient genetic model reveals regeneration from an *Insl3*-negative precursor population

The regenerative capacity of the ALC population has been recognized for decades through studies using EDS in rats (27-32). Although these studies provided fundamental insights into Leydig cell regeneration, EDS has important limitations. EDS displays marked species specificity, characterized by its lack of efficacy in murine models (29, 34, 42-44), and is further associated with systemic toxicity (18-25). Moreover, EDS predominantly eliminates mature Leydig cells while sparing immature and progenitor populations (26, 27, 32, 33, 45), making it difficult to determine whether regeneration results from differentiation of surviving committed cells or from activation of a more primitive precursor compartment. Despite its considerable value as a tool for investigating Leydig cell biology, the utility of EDS is constrained by its inability to act in mice, preventing its integration with the many gene knockout models developed for this species. Genetic approaches could overcome these limitations, but until recently suitable Leydig cell-specific Cre lines were unavailable.

A major advantage of the present model is the use of the highly specific *Insl3*^iCre^ allele, which targets all cells committed to the Leydig lineage, namely progenitor, immature, and mature ALCs. This contrasts with broader steroidogenic cell drivers such as *Cyp17a1*-Cre, which is known to target several tissues and cell types in addition to Leydig cells (reviewed in Ref. (46)) and may result in significant toxicity when coupled to DT-mediated ablation. In contrast, DT administration in *Insl3*^iCre^*;Rosa26*^TdTomato/iDTR^ mice was well tolerated and produced efficient Leydig cell depletion, allowing complete regeneration to be monitored over time.

The regeneration observed in our model provides strong functional evidence for the existence of an *Insl3*-negative precursor population. Because DT treatment eliminates progenitor, immature, and mature *Insl3*-expressing Leydig cells, regeneration cannot be attributed to surviving differentiated cells. Instead, newly formed TdTomato-positive Leydig cells must arise from precursor cells located upstream of *Insl3* activation (Fig. 10). Not only are these findings consistent with previous developmental and regeneration studies in rats that proposed the existence of SLCs in the postnatal and adult testis (reviewed in Refs. (2, 11, 12, 47, 48)) but they provide direct functional evidence in a mouse model following complete genetic ablation of the committed Leydig cell lineage. Thus, our model uniquely combines lineage specificity, efficient cell ablation, animal survival, and permanent fluorescent labeling of regenerated Leydig cells allowing direct visualization of the entire regenerative process.

### Regeneration originates from an *Insl3*-negative Leydig precursor population

Accumulating evidence suggests that ALCs arise from a population of stem or progenitor cells that persists beyond puberty and remains capable of responding to physiological and experimental challenges. Once activated, these cells follow a developmental progression in which SLCs differentiate through progenitor and immature intermediates before becoming fully mature testosterone-producing ALCs (reviewed in Refs. (2, 11, 48)). Leydig cell regeneration following EDS treatment was shown to recapitulate the developmental dynamics observed during normal postnatal Leydig cell maturation in rats ((49, 50), and reviewed in Ref. (13)).

Our findings provide functional support for this model. Following complete ablation of all detectable *Insl3*-positive Leydig cells, a new TdTomato-positive population reappeared and eventually repopulated the interstitial compartment. Because newly differentiated Leydig cells activate the *Insl3*^iCre^ allele and permanently express TdTomato (9), the regenerated population could be followed throughout the regenerative process. The appearance of a completely new TdTomato-positive Leydig cell population after disappearance of the original population strongly supports the existence of regenerative *Insl3*-negative precursors that survive DT-mediated ablation and subsequently enter the Leydig cell differentiation pathway.

The developmental framework described by Ademi et al provides important context for these findings (8). Steroidogenic progenitors are specified early during fetal development and express markers including COUP-TFII/NR2F2, ARX, and WNT5A (8). Their developmental potential evolves over time, with later progenitors acquiring characteristics of dormant ALC precursors and becoming closely related to PMCs (8). WNT5A lineage tracing further supports contributions to fetal and ALCs and PMCs (8). In our adult regeneration model, COUP-TFII-positive cells persisted immediately adjacent to the PMC layer after elimination of the *Insl3*-positive lineage and were αSMA-negative, CYP17A1-negative, and TdTomato-negative. Some COUP-TFII-positive cells at the peritubular surface subsequently began to acquire TdTomato fluorescence, consistent with transition from an *Insl3*-negative precursor state toward Leydig cell commitment. These observations link the adult regenerative population to the developmental steroidogenic progenitor framework while recognizing that formal lineage tracing will be required to establish stem cell identity.

### The peritubular compartment emerges as a major regenerative niche for ALC formation

The most striking biological finding of this study is the spatial distribution of newly forming Leydig cells during regeneration. The earliest TdTomato-positive cells appeared predominantly adjacent to αSMA-positive PMCs surrounding seminiferous tubules and, to a lesser extent, near perivascular cells associated with blood vessels. These emerging cells also expressed VCAM1, a marker associated with testicular mesenchymal progenitors (40).

Previous studies have implicated both peritubular and perivascular regions as potential reservoirs of Leydig progenitors (51-55). Our findings extend these observations by providing direct evidence during an active regenerative process. Rather than emerging randomly throughout the interstitium, newly differentiating Leydig cells were consistently associated with these anatomical niches, particularly the peritubular compartment. This suggests that the peritubular region functions as a major regenerative niche that supports precursor activation and Leydig cell differentiation.

The mechanisms responsible for this spatial organization remain unclear. PMCs may provide extracellular matrix components, structural support, or locally acting signals that promote precursor cell activation and differentiation. Alternatively, Leydig cells and PMCs may share developmental relationships through common mesenchymal progenitors. Recent transcriptional and lineage studies in humans, mice, and rats support close developmental links between these cell populations (6, 8, 35, 56-58). Future lineage-tracing experiments targeting candidate progenitor populations will be required to identify the precise cellular source of regenerated Leydig cells.

Although the signals that reactivate resident SLCs following ALC ablation remain poorly characterized, platelet-derived growth factor (PDGF) and Hedgehog signaling are particularly compelling candidates (reviewed in Refs. (11, 47, 48)). PDGF-A/PDGF receptor α (PDGFRA) signaling is required for normal ALC development in mice, while PDGF-AA promotes SLC differentiation (57, 59-61). Interestingly, resident stem cells located adjacent to PMCs can adopt a Leydig fate in response to PDGF-AA/LH or a myoid fate in response to PDGF-BB/TGFβ (57, 61). Desert Hedgehog (DHH) signaling also promotes SLC proliferation and commitment (55), whereas TGFβ and Notch signaling can maintain progenitor states or inhibit Leydig cell differentiation (55, 62). Thus, ALC loss may alter the balance of local signals within the peritubular niche, shifting resident stem cells from quiescence toward Leydig cell differentiation.

### Regeneration restores endocrine function and is accompanied by macrophage recruitment

Leydig cell ablation resulted in an almost complete loss of intratesticular and circulating testosterone, accompanied by rapid regression of androgen-dependent organs. As regeneration progressed, testosterone levels gradually recovered and ultimately returned to control values, paralleling restoration of seminal vesicle and epididymal weights (Fig. 10). The initial recovery of testosterone at day 14 coincided with the appearance of CYP17A1-positive regenerating Leydig cells and is consistent with the progressive acquisition of steroidogenic capacity during postnatal Leydig cell differentiation in mice (10). These findings demonstrate that regenerated Leydig cells acquire functional steroidogenic capacity and effectively restore endocrine homeostasis. Although testosterone production was restored, future studies will be required to determine whether regenerated Leydig cells fully recapitulate the molecular identity of native ALCs.

DT-mediated Leydig cell ablation was accompanied by activated Caspase 3 in TdTomato-positive Leydig cells, directly demonstrating apoptosis of the targeted population. This is consistent with established DT-mediated cytotoxicity following inhibition of protein synthesis (38, 63). A transient macrophage response accompanied this cell loss. This is consistent with the established functional relationship between macrophages and Leydig cells (reviewed in Refs. (41, 64)). Macrophages remain associated with newly emerging Leydig cells during early regeneration before returning to baseline levels once regeneration was complete (Fig. 10). The transient nature of macrophage recruitment observed in the present study raises the possibility that macrophages contribute actively to the regenerative process itself. Although our findings do not establish a direct role for macrophages in Leydig cell regeneration, the close temporal correspondence between macrophage recruitment and the appearance of newly formed Leydig cells suggests that macrophages may constitute a key element of the regenerative microenvironment. Their involvement may include remodeling of the interstitial niche and producing signals that support regeneration, consistent with observations from earlier studies in rats (65-67). Similar functions have been described in regenerative processes throughout multiple tissues, where macrophages coordinate debris clearance, extracellular matrix remodeling, and stem cell activation (reviewed in Refs. (68-70)).

In conclusion, the present study establishes a robust genetic model for complete Leydig-lineage ablation and regeneration in mice. Our results demonstrate that adult testes contain an *Insl3*-negative regenerative precursor population capable of restoring the entire Leydig cell lineage following injury. The preferential emergence of newly differentiating Leydig cells adjacent to PMCs identifies the peritubular compartment as a major regenerative niche and provides a framework for future studies aimed at defining the cellular and molecular mechanisms governing Leydig cell regeneration (Fig. 10).

## Data Availability

All data generated or analyzed during this study are included in this published article.
